# Faster, sharper, more precise: Automated Cluster-FLIM in preclinical testing directly identifies the intracellular fate of theranostics in live cells and tissue

**DOI:** 10.7150/thno.42581

**Published:** 2020-05-15

**Authors:** Robert Brodwolf, Pierre Volz-Rakebrand, Johannes Stellmacher, Christopher Wolff, Michael Unbehauen, Rainer Haag, Monika Schäfer-Korting, Christian Zoschke, Ulrike Alexiev

**Affiliations:** 1 Institute of Experimental Physics, Freie Universität Berlin, Arnimallee 14, 14195 Berlin, Germany; 2 Institute of Pharmacy (Pharmacology and Toxicology), Freie Universität Berlin, Königin-Luise-Str. 2+4, 14195 Berlin, Germany; 3 Institute of Chemistry and Biochemistry, Freie Universität Berlin, Takustr. 3, 14195 Berlin, Germany

**Keywords:** fluorescence lifetime imaging microscopy, high throughput imaging, FLIM automatization, nanomedicine, drug development

## Abstract

Fluorescence microscopy is widely used for high content screening in 2D cell cultures and 3D models. In particular, 3D tissue models are gaining major relevance in modern drug development. Enabling direct multiparametric evaluation of complex samples, fluorescence lifetime imaging (FLIM) adds a further level to intensity imaging by the sensitivity of the fluorescence lifetime to the microenvironment. However, the use of FLIM is limited amongst others by the acquisition of sufficient photon numbers without phototoxic effects in live cells. Herein, we developed a new cluster-based analysis method to enhance insight, and significantly speed up analysis and measurement time for the accurate translation of fluorescence lifetime information into pharmacological pathways.

**Methods**: We applied a fluorescently-labeled dendritic core-multishell nanocarrier and its cargo Bodipy as molecules of interest (MOI) to human cells and reconstructed human tissue. Following the sensitivity and specificity assessment of the fitting-free Cluster-FLIM analysis of data *in silico* and *in vitro*, we evaluated the dynamics of cellular molecule uptake and intracellular interactions. For 3D live tissue investigations, we applied multiphoton (mp) FLIM. Owing to Cluster-FLIM's statistics-based fitting-free analysis, we utilized this approach for automatization.

**Results**: To discriminate the fluorescence lifetime signatures of 5 different fluorescence species in a single color channel, the Cluster-FLIM method requires only 170, respectively, 90 counts per pixel to obtain 95% sensitivity (hit rate) and 95% specificity (correct rejection rate). Cluster-FLIM revealed cellular interactions of MOIs, representing their spatiotemporal intracellular fate. In a setting of an automated workflow, the assessment of lysosomal trapping of the MOI revealed relevant differences between normal and tumor cells, as well as between 2D and 3D models.

**Conclusion**: The automated Cluster-FLIM tool is fitting-free, providing images with enhanced information, contrast, and spatial resolution at short exposure times and low fluorophore concentrations. Thereby, Cluster-FLIM increases the applicability of FLIM in high content analysis of target molecules in drug development and beyond.

## Introduction

Drug development struggles with the identification of future medicines as reflected by success rates of clinical trials between 3% in oncology and 34% in vaccination 1. Current approaches to increase the success rate of investigational new drugs include the use of human cells, the reconstruction of 3D tissues, improved preclinical study design, and advanced analytical techniques such as omics or high content screening (HCS), the latter also named high content analysis or high content imaging 2-5.

High content analysis aims to contribute relevant information - from the identification of drug targets, fundamental biological processes and disease mechanisms, to safety pharmacology - by measuring multiple physiologically relevant features in cells, tissues, or organisms at once 5. High content imaging requires an automated, microscope-based analysis of the fluorescence intensity read-out of target molecules and the related cellular processes, including complex cellular effects and pathways.

Compared to the conventional fluorescence intensity-based microscopy, fluorescence lifetime based approaches offer several advantages. The fluorescence lifetime is independent of the fluorophore concentration and highly sensitive to the physicochemical environment, including temperature, pH, viscosity, polarity, or specific molecular interactions 6-9. Thus, fluorescence lifetime imaging microscopy (FLIM) has gained increasing interest for studying cellular processes 10-15. Recent applications of FLIM include the visualization of drug and drug delivery systems interacting with cellular targets as well as their transport and activation mechanism 16-24; even the capability for high content analyses was shown 25-27.

However, quantitative FLIM image analysis as needed for high content screening struggles with distinguishing the different fluorescence lifetime features of the exogenous and endogenous fluorophores because of insufficient photon counts per image pixels. As an example, different cellular drug binding modes can be distinguished by their local fluorophore environments, which are represented by a characteristic fluorescence decay. These characteristic fluorescence decays (fluorescence lifetime signatures, FLS) may contain a multitude of decay components. More than 10^4^ photons are required to accurately fit a biexponential fluorescence decay 28. Fast acquisition times, high magnification levels (subcellular resolution) and phototoxicity, all limit the detected photon number. Tau plot FLIM analysis commonly uses local pixel binning to increase photon counts for a more reliable fitting result at the expense of resolution, contrast, and lifetime information; this holds true in particular for multi-exponential fluorescence decays or lifetime distributions 29-32. Advanced FLIM analysis techniques include rapid global fitting (FLIMfit) 31, Laguerre expansion techniques for global fitting 33, Bayesian estimation 29, noise-corrected principal component analysis 34, pattern‐matching using reference fluorescence decay signatures combined with additional spectral information 35, or graphically based analyses, such as the phasor approach 29, 35, 36. Still, the major challenges for automated, fast, and reliable extraction of FLS in high content screening are not solved: (1) Discrimination of a multitude of fluorescent species with multi-component decay behaviour, and (2) automated output. The graphically based phasor plot can yield lifetime values for mono- and biexponential decays. FLIMfit is very fast for large data sets, but in essence is also limited to biexponential decays 31. The same holds true for Baysian estimation 37 and Laguerre expansion technique 33. Noise-corrected principal component analysis as described in 34 is not suitable for automated output. A reference-based pattern matching approach is not applicable for the situation of unknown fluorescence decay signatures 38. Thus, there is a need for an automated FLIM analysis, which can handle low photon counts while retaining the fluorescence lifetime information to provide relevant and complex biological data at fast acquisition times 39, and which increases the speed of analysis.

To meet this challenge in high content imaging, we present here a fast FLIM analysis tool that is based on a multivariate clustering method and does not rely on any *a priori* information. The method retrieves the fluorescence decay curves of individual fluorescent species without fitting, thereby distinguishing many different and multi-exponential fluorescence decay curves with a high dynamic range. The fitting-free approach offers the opportunity for automatization and increased accessibility for scientists, e.g., in drug development.

We demonstrate the potential applicability for high-content screening by the visualization of the spatiotemporal intracellular fate of molecules of interest (MOI). We use the interaction of a cargo-loaded dendritic core-multishell nanocarrier (NC) 40 with human skin cells as an example, both in 2D cell cultures and 3D skin models. This novel Cluster-FLIM tool will enable researchers to obtain a detailed insight into pharmacodynamics and pharmacokinetics of drug candidates, and to make informed decisions within the drug development process on a higher level of knowledge.

## Materials and Methods

**Materials.** Bodipy™493/503 (4,4-difluoro-1,3,5,7,8-pentamethyl-4-bora-3a,4a-diaza-s-indacene, Bodipy), LysoTracker™ Deep Red (LysoTracker), Cholera Toxin Subunit B - Alexa Fluor™647 Conjugate (CTB-A647) and CellMask™ Deep Red Plasma Membrane Stain (CellMask) were purchased from Thermo Fisher Scientific (Waltham, USA). 4′,6-diamidin-2-phenylindol (DAPI) was obtained from Dianova (Hamburg, Germany). Methyl-β-cyclodextrin (MβCD), genistein, wortmannin, fucoidan, polyinosinic acid (Poly I), and polycytidylic acid (Poly C) were obtained from Sigma Aldrich (München, Germany). Indocarbocyanine (ICC) was obtained from Mivenion (Berlin, Germany). A Caveolin-1-Alexa Fluor™488 (Cav-1-A488) conjugated antibody (reactivity: human, Clone# 7C8, Catalog# IC5736G) was obtained from R&D Systems (Minneapolis, Minnesota, USA). All other chemicals were of the highest purity available. 35 mm glass bottom cell culture dishes were purchased from Greiner Bio-One (Frickenhausen, Germany).

**Core-multishell nanocarrier synthesis and indocarbocyanine (ICC)-labeling.** The ICC-labeled core-multishell nanocarrier (NC-ICC) was synthesized as described 41. In short, hyperbranched polyglycerol amine (hPG-NH_2_) with a molecular weight of 10 kDa and a degree of functionalization of amines of 70% was reacted with approx. 1 molecule of a NHS-ester of the ICC dye. Afterwards, the residual amines were reacted with the amphiphilic double shell, resulting in the empirical formula PG_10000_ (NH_2_)_0.7_(C_18_mPEG_7.2_)_0.98_(ICC_0.02_). The cargo Bodipy has a logP value of 3.50 ± 0.04 (octanol/water) 42. Encapsulation by the nanocarriers was performed using a variation of the so-called film uptake method 41. 1.2 mg of Bodipy was dissolved in ethanol, added into a vial and the solvent evaporated, leaving a film of the dye. The aqueous nanocarrier solution (1.5 mL, 5 g/L) was then added and the suspension stirred for 22 h at 1200 rpm and filtrated (regenerated cellulose, 450 nm pore size). The amount of encapsulated Bodipy was determined to 0.0027% (0.7 molecule Bodipy per molecule NC-ICC) by absorption spectroscopy after lyophilisation and redissolution of an aliquot in methanol using the extinction coefficient of Bodipy ε = 91000 M^-1^cm^-1^ at 493 nm (**SI Figure S1**).

**Cell and tissue culture.** Normal human keratinocytes and normal human dermal fibroblasts were isolated from juvenile foreskin of medically-indicated circumcisions of boys younger than 9 years old. Primary keratinocytes and fibroblasts (passage 3, pooled from three donors) were from therapeutically indicated circumcisions (ethical approval EA1/081/13, ethics vote from the Charité-Universitätsmedizin Berlin) after parents had signed the written informed consent. The SCC-25 cell line, passage 98-100, were obtained as a gift from Howard Green (Dana-Farber Cancer Institute, Boston, MA, USA) and were authenticated by single nucleotide polymorphism profiling (Multiplexion, Heidelberg, Germany). For all 2D live-cell FLIM experiments 2.5 x 10^5^ cells were seeded per compartment of glass bottom cell culture dishes and cultured in their respective medium for 2 days. For keratinocytes, Keratinocyte Growth Medium (KGM, Lonza, Köln, Germany) was used. Cell culture was performed according to standard operating procedures and referred to good cell culture practice.

SCC-25 were cultured in DMEM/F12, supplemented with 100 U/mL Penicillin, 100 µg/mL Streptomycin (Sigma Aldrich, München, Germany) and 2 mM L-Glutamine. Media and supplements were purchased from Sigma Aldrich, München, Germany; media were changed once on the 2^nd^ day.

3D skin tissue models were grown as described previously 43, 44.

**2D and 3D uptake studies.** Keratinocyte cells (2.5 x 10^5^ cells per compartment) were incubated for 15, 45, 90, 180, and 270 minutes at 37 °C and 5% CO_2_ with NC-ICC/Bodipy at a concentration of 10 µg/mL in KGM. Directly before FLIM measurements (see below), cells were washed twice and KGM was exchanged with PBS (pH 7.4). Subsequently, the cells were stained with DAPI (28 µM) and CellMask (0.5 µg/mL) for 5-10 minutes. FLIM experiment series were performed four times. For each experiment series, and each time point two different images (usually full image and zoom) were recorded.

SCC25 cells were incubated in the respective growth medium (see above) for 15, 180, 270, 370 min. Cell uptake of NC-ICC was directly investigated by FLIM after two washing steps and medium exchange to PBS (pH 7.4).

For 3D skin model uptake studies, 30 µL/cm^2^ of NC-ICC in PBS (pH 7.4) was applied onto the tissue surface for 6 and 22 h at 37°C, 5% CO_2_. The incubated sample was placed top down (with the *stratum corneum* facing the bottom) into a 35 mm glass bottom cell culture dish and humidified by filter paper soaked with PBS before subjected to mpFLIM. For reference cryosections, the 3D skin models were snap frozen and sectioned into 7-µm slices (Leica CM 1510S, Wetzlar, Germany).

**Fluorescence lifetime imaging microscopy.** Fluorescence lifetime imaging microscopy (FLIM) was performed in a home-built setup 45, 46. The setup consists of an inverted microscope (IX71, Olympus, Shinjuku, Tokyo, Japan), a tunable ps-supercontinuum white light laser (SuperK Extreme EXU-3, NKT Photonics, Birkerød, Denmark), a ps-diode laser (BDL-405-SMN), a confocal scanning unit (DCS120), a hybrid PMT detector (HPM-100-40), and a time-correlated single photon counting (TCSPC) module (SPC160, all Becker & Hickl, Berlin, Germany). FLIM images were recorded by the SPCM software (Becker & Hickl, Germany) using a 60x objective (water, UPLSAPO60XW, Olympus, Japan) resulting in a total field of view with a side length of 300 µm. An acoustic-optical tunable filter (SELECT UV-VIS, NKT Photonics, Denmark) was used to select the individual fluorescence excitation wavelengths from the white light laser beam. The laser repetition rate was set to 19.5 MHz. Bodipy and Cav-1-A488 fluorescence were excited at 488 nm, NC-ICC fluorescence at 530 nm, and CellMask, CTB-A647 as well as LysoTracker fluorescence at 640 nm. DAPI fluorescence was exited at 405 nm by a ps-diode laser (BDL-405-SMN, Becker & Hickl, Germany) at a repetition rate of 20 MHz. Time-resolved fluorescence emission was spectrally selected by a band-pass filter (525/50 nm, Semrock, Rochester, USA) for Bodipy and Cav-1-A488, a combination of a long-pass filter (>545 nm, Chroma, Rockingham, USA) and a short-pass filter (<600 nm, Coherent, Santa Clara, USA) for CMS-ICC, a long-pass filter (>665 nm, Chroma, Rockingham, USA) for CellMask, LysoTracker, and CTB-A647 and a band-pass filter (452/45 nm, Semrock, USA) for DAPI. The TCSPC-module sorted the detected fluorescence photons into 1024 time channels with a channel width of 19.97 ps. The instrument response function of the system was less than 100 ps (FWHM). The acquisition time for Bodipy and NC-ICC was set to 300 s, for Cellmask and DAPI to at least 120 s. For live-cell applications, a temperature-controlled specimen holder was installed and adjusted to either 4°C or 37°C. Living cells were measured in glass bottom cell culture dishes (Greiner Bio-One, Germany).

**Multiphoton fluorescence lifetime imaging microscopy.** Multiphoton FLIM (mpFLIM) was conducted in a home-built setup 47. A mode-locked pulsed femtosecond Ti:sapphire laser (Mira 900, Coherent, USA) is pumped by a diode-pumped solid state laser (Verdi V5, Coherent, USA) generating laser pulses shorter than 200 fs with a repetition rate of 76 MHz at a wavelength of 800 nm. An objective (60x water, UPSLAPO60XW, Olympus, Japan) focused and a scanning unit (DCS-120, Becker & Hickl, Germany) scanned the excitation laser beam over the sample placed on an inverted microscope (IX-73, Olympus, Japan). Fluorescence emission was separated from excitation by a dichroic mirror (H 643 LPXR superflat, AHF, Germany) and a short-pass filter (SP745 BrightLine HC, Semrock, USA). NC-ICC fluorescence was distinguished from fluorescence of other fluorescent species by a combination of a long-pass filter (>545 nm, Chroma, USA) and a short-pass filter (<600 nm, Coherent, USA) generating a spectral detection window of 545 to 600 nm. Fluorescence photons were collected in non-descanned detection mode by a hybrid detector (HPM-100-40, Becker & Hickl, Germany). The IRF of the system was below 120 ps (FWHM). Collected photons were sorted into 1024 time channels (width 9.7 ps) by a TCSPC module (SPC-160, Becker & Hickl, Germany). The same cluster-based FLIM analysis as for one-photon FLIM was applied.

**Cluster-FLIM analysis.** The temporal decay characteristics of multicomponent TCSPC data from a fluorescence decay matrix, i.e., the fluorescence decays in each pixel of an image, and the Poisson distributed signal noise restrict the extraction of the underlying fluorescence decay curves 35. The underlying decay curves (FLSs) in the fluorescence decay matrix may originate from a multitude of fluorescing molecules and/or varying fluorescence decays of the same fluorophore depending on the local environment. The problem of determining the intrinsic unknown structure of the fluorescence decay data, when no information other than the recorded FLIM data is available, can be solved by grouping similar patterns (fluorescence decays) into clusters. The cluster can be discriminated from each other according to some similarity/dissimilarity measure used by a cluster algorithm, e.g., the Euclidian distance in *k*-means 48. No a priori knowledge of patterns (i.e. FLSs) that belong to certain groups is necessary for this type of clustering. The raw FLIM data can be described as a set of patterns *X' =* [*x'*_1_*,…, x'_i_,…, x'_N_*}, where *x'_i_* represents the fluorescence decay histogram *x'_i_ =* (*x'_i,_*_1_*,…, x'_i,j_,…, x'_i,b_*)^T^ constructed of *b* time bins in the single pixel *i* of the *N* pixels in a FLIM-image. The *d* dimensional feature space, with the individual features *x_i,j_*_,_ is generated and the Euclidian distance *D_i,j_* for each cluster member *x_i_* to a respective cluster center *x_c_* is calculated by


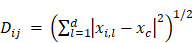
(1)

and serves as a similarity/dissimilarity measure in the clustering. Using the distances *D_i,j_* the fluorescence decay patterns of the individual pixels were partitioned into meaningful groups (i.e. clusters) by applying the *k*-means algorithm 48. A validation of the method is shown in **SI Figure S2.** False-color images were generated by assigning a distinct color to all pixels containing a fluorescence decay trace that belonged to a certain cluster. The FLS of an identified cluster is generated through accumulation of all photons from image pixels belonging to this cluster. Subsequently, the FLSs could be fitted by deconvolution of a multi-exponential model function with a calculated IRF. After deconvolution of the fluorescence traces and the IRF, and taking the background counts into account, the time-dependent decay profile was fitted to a multi-exponential model function described by

I(t)=Σ_i_^n^α_i_e^-t/τi^ (2)

with* n* the total number of decay components; α_i_ the amplitude and τ_i_ the fluorescence lifetime of the *i-*th component 49-52. The mean (amplitude weighted) fluorescence lifetime *τ_m,a_* was obtained from the following equation

τ_m,a_=Σ_i_^n^α_i_τ_i_ (3)

and the component weighted mean fluorescence lifetime *τ_m_* was calculated by

τ_m_= Σ_i_^n^β_i_τ_i_ (4)

with *β_i_* being the fractional amplitude of the *i-*th component with

β_i_=α_i_τ_i_/ Σ_i_^n^α_i_τ_i_ (5)

A detailed account on the use of average fluorescence lifetime is given in 52, 53. FLIM data were analyzed with self-written routines in C++.

Analytical qualification of the Cluster-FLIM analysis tool was determined for sensitivity (hit rate) and specificity (correct rejection rate) in accordance with International Council for Harmonisation of Technical Requirements for Pharmaceuticals for Human Use guidelines 54:

Specificity = true negatives/(true negatives+false positives) (6)

Sensitivity = true positives/(true positives+false negatives) (7)

**Spatiotemporal development of the FLIM-Clusters in keratinocytes and kinetic model of NC-ICC internalization and transport.** For analyzing the spatiotemporal development of the FLIM clusters in NHK cells we performed kinetic modelling using the relative cluster concentrations. The relative cluster concentrations were expressed as the fraction of the total NC-ICC intensity. The time-dependence of the concentration of the three FLIM-clusters (cyan (c), yellow (y), red (r)) in the plasma membrane (pm) and in the cytoplasm (cp) was fitted to a simple kinetic reaction model:

[NC-R]_pm,c_ ⇌ [NC-R-C]_pm,y_ → [NC-R-C]_cp,y_ → [NC*]_cp,r_ (8)

with [NC-R]_pm,c_ being NC-ICC bound to SR on the plasma membrane (cyan cluster). [NC-R]_pm,y_ describes NC-ICC bound to SR in lipid raft/caveolae within areas of the plasma membrane (yellow cluster). [NC-R-C]_cp,y_ is the fraction of NC-ICC bound to SR in caveolar vesicles in the cytoplasm (yellow cluster), while NC*_cp,r_ describes NC-ICC in lysosomal compartments (red cluster). We assume first order reactions and describe the kinetic model of NC-ICC internalization and transport by the following set of differential equations:


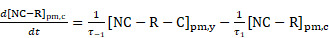
(9)


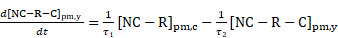
(10)


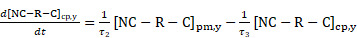
(11)


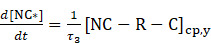
(12)

where *τ*_1_ is the time constant of the transition of receptor bound NC-ICC in the plasma membrane to lipid raft/caveolae containing membrane areas. *τ*_-1_ describes the time constant of the corresponding back transition. *τ*_2_ is the time constant of the dissociation of NC-ICC loaded caveolar vesicles from the plasma membrane into the cytoplasm and *τ*_3_ describes the time constant of the transport into lysosomes. The fitting procedure with the differential equations (Eq. 9-12) was conducted in Mathematica 11.0 (Wolfram Research, Champaign, IL, USA).

**Concentration dependent NC-ICC uptake in monolayer experiments and displacement of NC-ICC from keratinocyte cell membranes by NC.** To investigate the concentration dependence of the NC-ICC uptake, keratinocytes were incubated with NC-ICC for 4.5 h at concentrations ranging from 0 to 25 µM in KGM at 37°C. The uptake behavior was analyzed by the intracellular fluorescence intensities at the different NC-ICC concentrations. The observed non-linear uptake behavior was fitted by a Michaelis-Menten-like equation 55:

I=I_max_*[NC-ICC]/(K_50_+[NC-ICC]) (13)

with *I*_max_ being the saturation intensity, [NC-ICC] the concentration of NC-ICC, and *K_50_* the half-maximum uptake concentration.

To study NC-ICC displacement from the cell membrane by unlabeled NC, monolayer cultures were incubated with NC-ICC (0.5 µM) in KGM at 4 °C for 1 h. Afterwards, unlabeled NC was added in concentrations ranging from 10 to 10000 nM at 4 °C. The displacement of NC-ICC from the cell membrane was analyzed using the NC-ICC occupied cell membrane area. The decrease of occupied membrane areas with increasing NC concentrations was fitted by a modified Hill-function:

A=A_max_+((A_min_-A_max_)[NC]^n^)/(K_50_^n^+[NC]^n^) (14)

where *A_max_*, *A_min_* are the maximal and minimal area of NC-ICC membrane occupation, [NC] the NC concentration, *K_50_* the half-maximum binding constant (apparent binding affinity), and *n* the Hill-coefficient (cooperativity factor).

**Inhibition studies of cellular uptake of NC-ICC towards primary keratinocytes**. Uptake inhibition and receptor blocking experiments were performed by a 30 to 60-minute pre-incubation with the respective inhibitor and a subsequent co-incubation with NC-ICC/Bodipy without any washing or changing of KGM at 37°C. For ATP inhibition cells were incubated with azide (3 mg/mL). Cholesterol depletion experiments were performed by incubation with methyl-β-cyclodextrin (5 mg/mL). Phosphoinositide 3-kinase activity was inhibited by wortmannin (150 ng/mL). Clathrin-mediated endocytosis was inhibited by chlorpromazine (10 µg/mL). Caveolae-mediated cell uptake was blocked by genistein (27 µg/mL). Scavenger receptor (SR) binding was blocked by polyinosinic acid (50 µg/mL) or fucoidan (100 µg/mL). As a positive control, keratinocyte cells were incubated with polycytidylic acid, not a ligand of SR. Low temperature experiments were performed at 4°C. For investigating binding of unlabeled NC to SR of normal keratinocytes, cells were incubated at 4°C with NC at a concentration of 1000 µg/mL. Subsequently, cells were incubated for at least 180 minutes with NC-ICC/Bodipy at a concentration of 10 µg/ml in KGM.

**Co-localization studies of NC-ICC with cholera toxin subunit B, caveolin-1 and LysoTracker.** For co-localization with cholera toxin subunit B uptake pathways, normal keratinocytes were incubated for 15 min with NC-ICC/Bodipy (10 µg/mL) and CTB-A647 (5 µg/mL) at 37°C and 5 % CO_2_ in KGM. For co-localization with caveolae, cells were incubated for 90 min with NC-ICC (10 µg/mL) and a Cav-1-A488 antibody at a 1:20 dilution. For lysosomal co-localization studies, cells were incubated for 600 min with NC-ICC/Bodipy (10 µg/mL) and during the last 45 minutes LysoTracker was co-incubated at a concentration of 50 nM. FLIM measurements were conducted without any washing or exchanging of KGM. Co-localization of NC-ICC and cholera toxin subunit B, Caveolin-1 and LysoTracker was quantified by Manders' co-localization coefficients M_1_ and M_2_, after an automatic threshold search as described in 56. M_1_ and M_2_, representing the overlapping fraction of two spectral channels A and B with respect to the other, i.e., M_1_ is the overlap of spectral channel A with spectral channel B and M_2_ is the overlap of spectral channel B with spectral channel A. Manders' co-localization coefficients can take values between 0 (no co-localization) and 1 (co-localization). To obtain the coefficients we used the Coloc2 plugin integrated in the image-processing package FIJI 57.

## Results and Discussion

Cluster-FLIM principle, performance, and cellular interactome of MOIs. We present a fast, statistics-based FLIM approach using multivariate cluster analysis 58. Cluster-FLIM solves the problem of determining the intrinsic unknown structure of the fluorescence decay data to reliably distinguish many characteristic fluorescence decay curves (from different fluorescent species or fluorophores) without fitting the lifetime data. As an example in the context of drug development, the Cluster-FLIM approach utilizes the influence of the microenvironment of MOIs to identify intracellular binding. Each binding event is characterized by its specific fluorescence decay curve, which constitutes a unique fluorescence lifetime signature (FLS).

**Figure 1:**
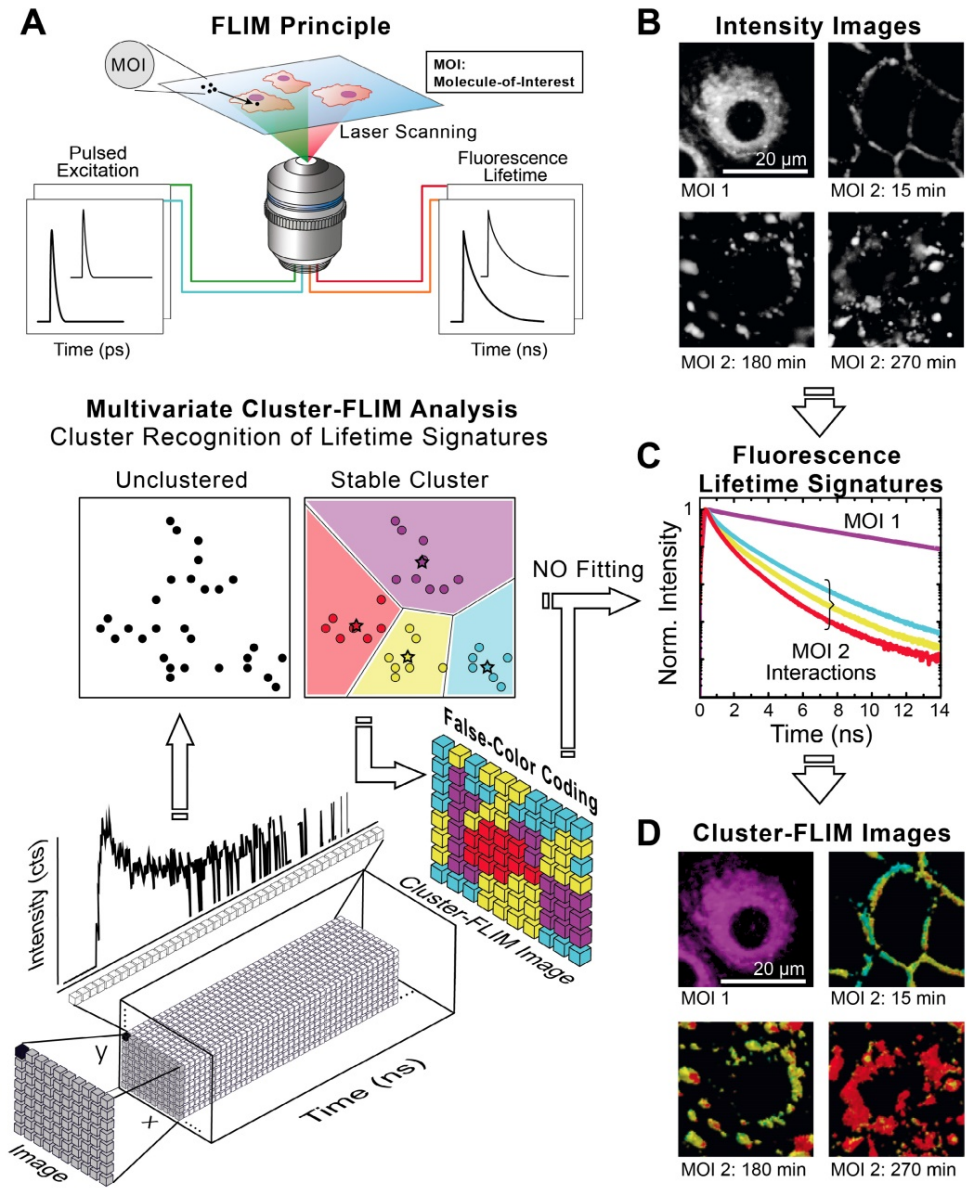
Cluster-FLIM principle and application for live cells. (**A**) FLIM principle and scheme of Cluster-FLIM analysis. False color coding of the images is based on cluster recognition of the different fluorescence lifetime signatures obtained from the FLIM data stacks. (**B**) Intensity images of MOIs in living human keratinocytes. (**C**) Fluorescence lifetime signatures (FLS) of MOI 1 (cargo Bodipy, magenta) and MOI 2 (NC-ICC, cyan, yellow, red). (**D**) Cluster-FLIM images as in (**B**), color-coded according to the FLS in (**C**). Experimental conditions for ICC: λ_ex_=530 nm, λ_em_=545-600 nm; for Bodipy: λ_ex_=488 nm, λ_em_=500-550 nm, measurements at 37°C.

**Figure 1A** shows schematically the principle of Cluster-FLIM and its application in live-cell studies. Several fluorescent MOIs can be excited simultaneously by a white-light supercontinuum laser system as a multicolor picosecond excitation source for TCSPC 45 (**Figure 1A**, upper part). Multivariate Cluster-FLIM (**Figure 1A**, middle and lower part) groups, e.g., the ~ 260.000 fluorescence decay curves in the individual pixels of a single image with 512x512 pixels according to their similarities in the fluorescence decay characteristics into stable clusters (**Figure 1A**, middle part). The similarity in this example is visualized by color-coding of the different clusters (red, yellow, cyan), each representing a group with a characteristic FLS (e.g. **Figure 1C**). The color code is then used for false-color coding of the image (**Figure 1A**, lower part). The representative fluorescence lifetime curve, FLS, of an identified cluster is generated through accumulation of all photons from image pixels belonging to this cluster and thus shows an inherently high signal-to-noise ratio (**Figure 1C**) that exceeds the low signal-to-noise statistics of an individual pixel (**Figure 1A**, lower part). Most importantly, the FLS is obtained without any fitting. Since the cluster approach directly refers the cluster identity back to the image pixel, it yields immediate resolution, contrast, and content enhancement (**Figure 1D**), not only compared to intensity-based images (**Figure 1B**) but also to the currently available FLIM analysis tools (**SI Figure S2B**).

We visualize the interactome of two MOIs, an indocarbocyanine dye (ICC, a Cy3 derivative) labeled dendritic core-multishell nanocarrier (in short NC-ICC) and its fluorescent cargo Bodipy (**SI Figure S1**), with normal human keratinocytes. MOI 1, Bodipy, is taken up within seconds to minutes into the cytoplasm without any further changes to its location (**Figure 1D**). For MOI 2, Cluster-FLIM identifies specific interactions of NC-ICC in these cells that are not static, but dynamically changing with incubation time, as visualized by the different colors (FLS) in **Figure 1D**. The FLSs of the extracted cluster from MOI 2 display multiexponential decay behavior (**Figure 1C, SI Table S1**).

Cluster-FLIM was validated using synthetic data (**SI Figure S2**) consisting of seven different FLS with multiexponential decays, three of which sharing the same mean lifetime. Compared to a conventional Tau plot, even low photon counts in a single pixel are sufficient to extract reliably multicomponent FLS (**SI Figure S2**). The difference between Cluster-FLIM and Tau plot for real experimental FLIM data reveals the increase in contrast and content by Cluster-FLIM (**SI Figure S2**).

**Figure 2:**
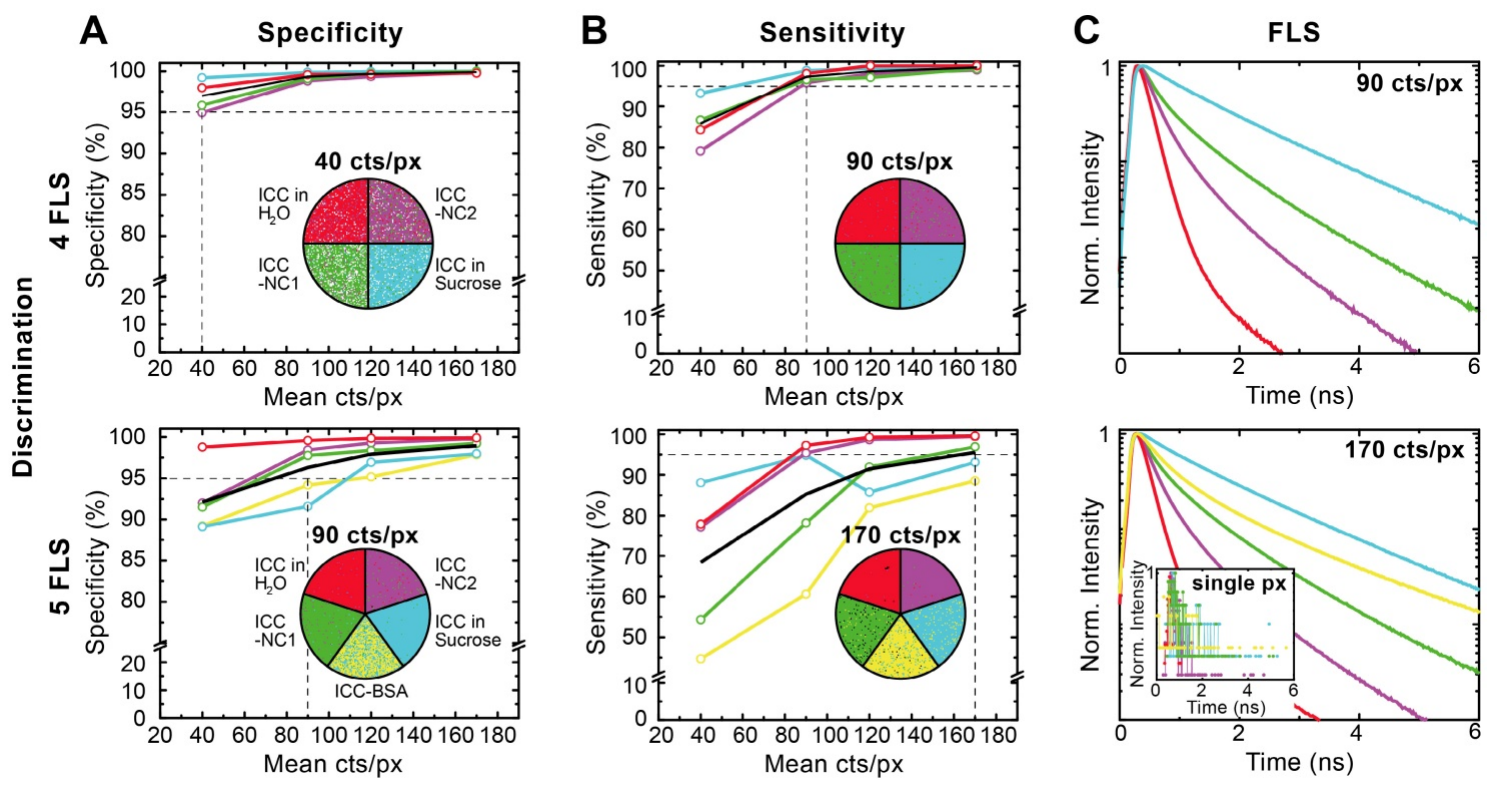
Validation of Cluster-FLIM for the fluorophore ICC in 4 and 5 different environments. (**A**) Specificity (correct rejection rate) and (**B**) sensitivity (hit rate) is calculated from experimental data as a function of mean pixel counts (40, 90, 120, 170 counts/pixel (cts/px)). Samples: ICC in H_2_O (red), in 69% sucrose (w/w) (cyan), attached to NC1 (green), to NC2 (magenta), and to bovine serum albumin (BSA) (yellow). Cluster-FLIM images for the respective mean pixel counts to obtain either 95% specificity or 95% sensitivity (indicated as the intersection of the horizontal and vertical dashed lines) are shown. White pixels in (**A**) are below a threshold of 10 cts/px and do not contribute to the Cluster-FLIM analysis. (**C**) Fluorescence lifetime signatures (FLS) for mean pixel counts at 95% sensitivity. Inset: Fluorescence decay in a single pixel (px) at 170 cts/px for comparison. SI Table S2 summarizes the different fluorescence lifetimes (mean fluorescence lifetime varies between 0.20 ns and 1.53 ns). Experimental conditions for ICC as in Figure 1.

To quantify the advantages of Cluster-FLIM, we evaluated the specificity and sensitivity of the method by measuring and analyzing the fluorescent dye ICC in four or five different microenvironments at once (**Figure 2**). The Cluster-FLIM method provides 95% specificity (**Figure 2A**) and 95% sensitivity (**Figure 2B**) for the discrimination of the fluorescence lifetime signatures of four different fluorescence species (here four different microenvironments) with photon numbers as low as 40 and 90 counts per pixel, respectively (**Figure 2**, top). In the case of five different fluorescent species (**Figure 2**, bottom) 95% specificity and sensitivity are obtained with 90 and 170 counts per pixel, respectively. **Figure 2C** shows the corresponding FLS. The inset in **Figure 2C** (bottom) presents the fluorescence decays with 170 counts in a single pixel, i.e., on average only every 6^th^ point on the time axis of the lifetime histogram contains one photon. No separation between the different fluorescent species is possible based on these data (**Figure 2C, inset**). This clearly demonstrates the advantages of the fitting-free Cluster-FLIM method for extraction of the multiexponential FLS with high signal-to-noise ratio at low photon counts. Thus, Cluster-FLIM enables accurate extraction of multiexponential FLS (**SI Table S2**) at fast acquisition times and at very low photon statistics per pixel compared to the 10^4^ photons required in accurate biexponential fitting-based approaches 28.

**Correlation of Cluster-FLIM results to classical pharmacological uptake pathway analysis.** The dynamic interaction of MOI 2 (NC-ICC) in human keratinocytes (**Figure 1D**) represents the intracellular MOI interactome, visualized by the different FLS. This MOI interactome obtained by Cluster-FLIM ideally represents all the interactions along the uptake pathway into the cell.

**Figure 3:**
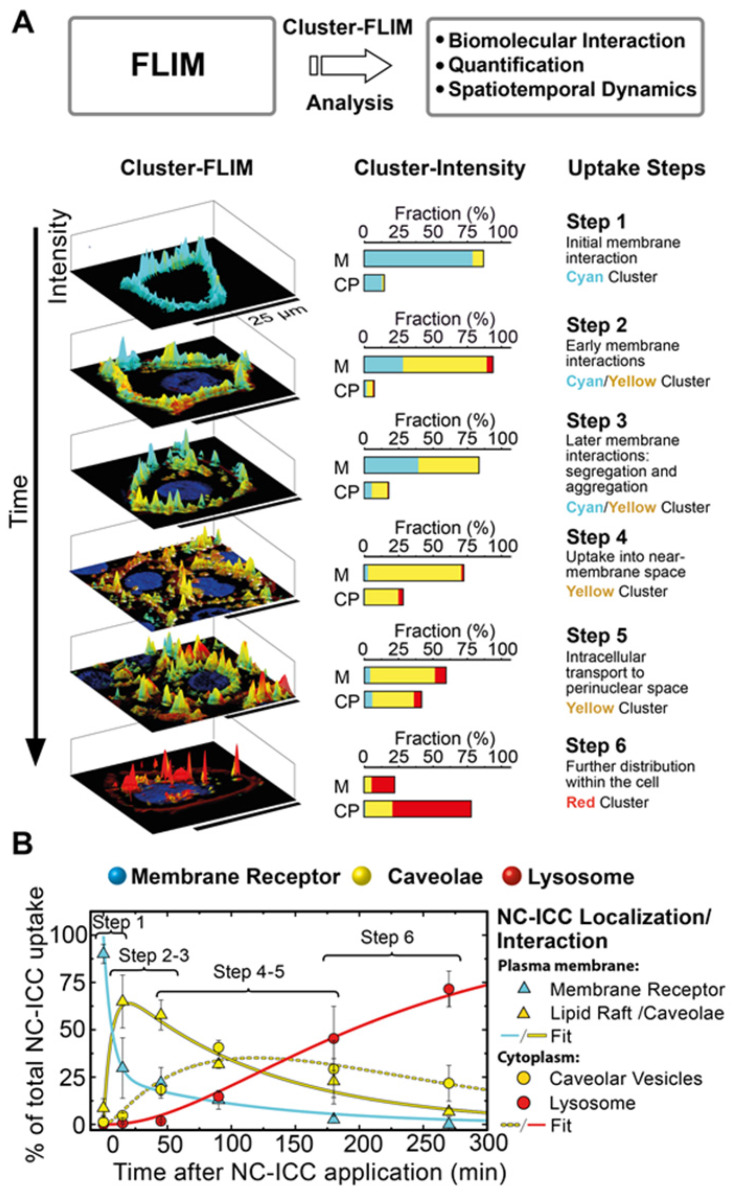
MOI interactome obtained by Cluster-FLIM. (**A**) Scheme and assessment of individual uptake steps of MOI 2 (NC-ICC) by amount and appearance of the FLIM clusters within the cell (cell membrane (M), cytoplasm (CP)). Nuclei are stained by DAPI (blue), plasma membranes by CellMask (dark red). Cyan, yellow, and red indicate the different FLIM clusters. (**B**) Temporal development of NC-ICC appearance. Values are mean ± SD. Fits according to the uptake and transport model (Eq. 8-12) are shown. Fit time constants: τ_1_ = 8 ± 2 min (membrane receptor → caveolae), τ_-1_ = 28 ± 11 min (back reaction of τ_1_ ), τ_2_ = 88 ± 6 min (internalization of receptor-NC-ICC complex by caveolae), τ_3_ = 107 ± 9 min (transport to lysosome).

To validate this assumption, we first analyzed the FLS distribution and intensity of the molecule of interest, NC-ICC, in live cells and categorized steps of cellular uptake and intracellular fate according to the spatio-temporal distribution of the respective cluster (**Figure 3**). The individual uptake steps were assessed solely by the amount and appearance of the FLIM clusters within the cell.

**Figure 4:**
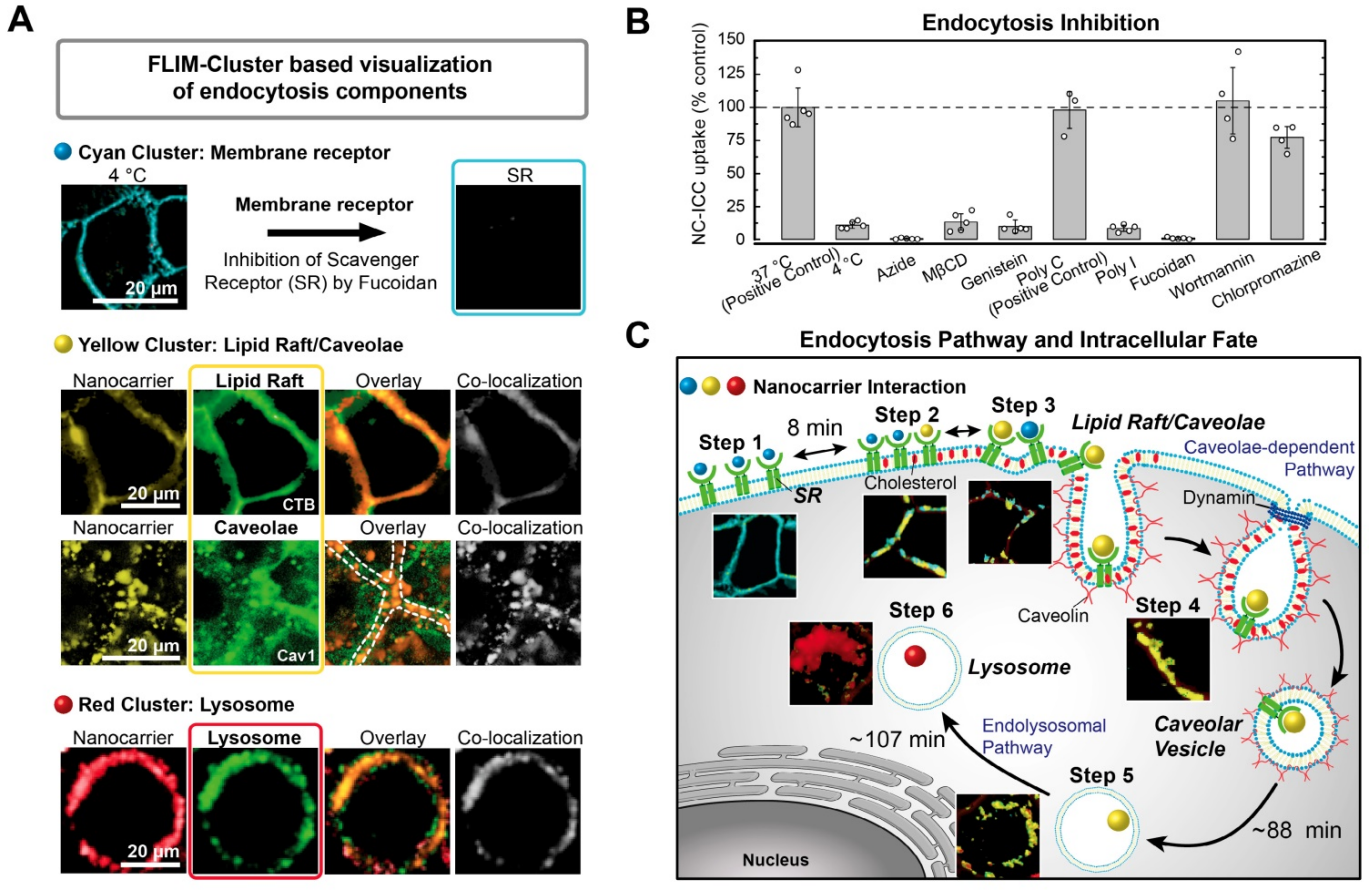
Unravelling of cellular pathways based on Cluster-FLIM interactome analysis. (**A**) Classical pharmacological uptake experiments as shown by imaging (inhibition and colocalization). Assignment of the cyan FLIM-cluster to scavenger receptor (SR) binding. Co-localization of yellow FLIM-cluster with lipid rafts stained by CTB-A647 and with caveolae stained by a caveolin-1 antibody (Cav-1-A488). Co-localization of red FLIM-cluster with lysosomes stained by LysoTracker. (**B**) Quantification of endocytosis inhibition. (**C**) Scheme of endocytosis pathway and intracellular fate deduced solely based on the Cluster-FLIM interactome analysis of MOI 2, the core-multishell nanocarrier (Figure 3). Experimental conditions as described in Materials and Methods.

Until 15 min after NC-ICC application, the cyan cluster depicts the dominating cluster and is observed almost exclusively at the cell membrane (**Figure 3A**). This effect occurs at both 37°C and 4°C, indicating passive binding (**Figure 4A, SI Figure S3**) and was labeled step 1 of the cellular NC-ICC interactome (**Figure 3A**). As the cyan FLS intensity decreases in the cell membrane, the cluster of the yellow FLS appears first in the membrane (step 2 and 3) and then shifts to the cytoplasm (step 4, **Figure 3A**). The appearance of the red cluster continuously increases from step 4 to step 6, forms the main cluster in step 6, and is predominantly located in the cytoplasm (**Figure 3A**).

Second, we performed classical pharmacological uptake experiments with NC-ICC to correlate the uptake steps identified from the Cluster-FLIM interactome to cellular pathways, for the case at hand, in normal human keratinocytes (**Figure 4**).

Cyan FLS: As low temperature strongly reduces the NC-ICC uptake into the cells but not its cell membrane binding, internalization of NC-ICC is an active process. An energy-dependent transport is confirmed by intracellular ATP-depletion using azide (**Figure 4B** and **SI Table S3**). Uptake of NC-ICC yields a saturation curve with increasing nanocarrier concentrations (**SI Figure S3**), which is expected for receptor-mediated uptake 55. Both the ICC tagged and the untagged NC bind to the same receptor as shown by inhibition and competition experiments (**SI Figure S3**). To identify the membrane receptor, we inhibited scavenger receptors (SRs), which belong to the cell-surface pattern-recognition receptors and bind a variety of ligands including nanocarriers 59. Poly I and fucoidan, well-known SR ligands, in particular for SR class A, inhibit NC-ICC uptake by more than 90% and 99%, respectively, while Poly C, not a ligand for SR 60, shows no inhibition (**Figure 4A,B**). Thus, we assign the cyan FLS to the interaction of the NC-ICC with a membrane receptor (SR) in the cell membrane.

Yellow FLS: The segregation/aggregation process observed in step 3 (**Figure 3A**) and the binding of NC-ICC to SR points to the involvement of lipid rafts 61, 62. While almost complete (~90%) inhibition of NC-ICC uptake is observed in the presence of MßCD and genistein as lipid raft/caveolae-mediated uptake inhibitors 63-65, blocking of macropinocytosis via the phosphoinositide 3-kinase inhibitor wortmannin 66 and clathrin-mediated endocytosis by chlorpromazine 67 does not affect NC-ICC uptake (**Figure 4B, SI Table S3**). Additionally, we demonstrated co-localization of the yellow FLIM cluster with the fluorescently labeled cholera toxin B (CTB-A647) 59, 68 and with a fluorescently tagged caveolin-1 antibody (**Figure 4A**). Co-localization was assessed by Manders' coefficients 56 with M_1_ = 0.59 and M_2_ = 0.53 for CTB-A647, indicating an interaction of NC-ICC with structures of caveolae/lipid rafts. The Manders' coefficients of M_1_ = 0.81 and M_2_ = 0.64 reveal very high co-localization of NC-ICC and caveolin both in the membrane as well as in the cytoplasm (**Figure 4A**). Taken together, the yellow FLS identifies the NC-ICC interaction with caveolae, both in the cell membrane and in the lumen of cells.

Red FLS: The red FLS appeared last (180 min - 270 min) and predominantly in the cytoplasm (**Figure 3**). Thus, we mapped the co-localization of the red FLIM-cluster with LysoTracker for prolonged (10 h) incubation times (**Figure 4A**) to unravel the intracellular fate. Manders' coefficients of M_1_ = 0.87 and M_2_ = 0.79 demonstrate a high co-localization of NC-ICC with LysoTracker. Thus, the red FLS indicates a lysosomal fate of NC-ICC.

The blocking and co-localization experiments agreed very well with the previously identified six uptake steps (**Figure 3A**). The uptake kinetics obtained directly from the Cluster-FLIM analysis (**Figure 3B**) are in accordance to a caveolae/lipid raft mediated uptake pathway (**Figure 4C**). Relatively long time constants are typical for the highly regulated caveolae/lipid raft mediated uptake process that involves complex cell signaling 63, 69, 70. In our case, NC-ICC is rapidly captured by caveolae in the cell membrane (8 ± 2 min). A back-reaction (28 ± 11 min) delays the NC-ICC internalization (88 ± 6 min). Similar half-lives of around 90 minutes were found for the prototypical internalization of the SV40 virus by caveolae 71. Eventually, the NC-ICC accumulates in lysosomes with a time constant of 107 ± 9 min.

Taken together, the inhibition and co-localization experiments proved the validity of Cluster-FLIM for unraveling cellular pathways (**Figure 4C**), solely based on the Cluster-FLIM interactome analysis (**Figure 3**). This lays the foundation for fast and automated decision making in drug development processes using Cluster-FLIM.

**Translating Cluster-FLIM interactome data to automated MOI assessment.** The encouraging results of MOI interactome visualization in live cells prompted us to test the analytical potential of Cluster-FLIM by quantifying interactome differences between various cell types. Using normal and tumor cells, we exemplify that the interactome assessment allows informed decision making for drug development processes.

**Figure 5:**
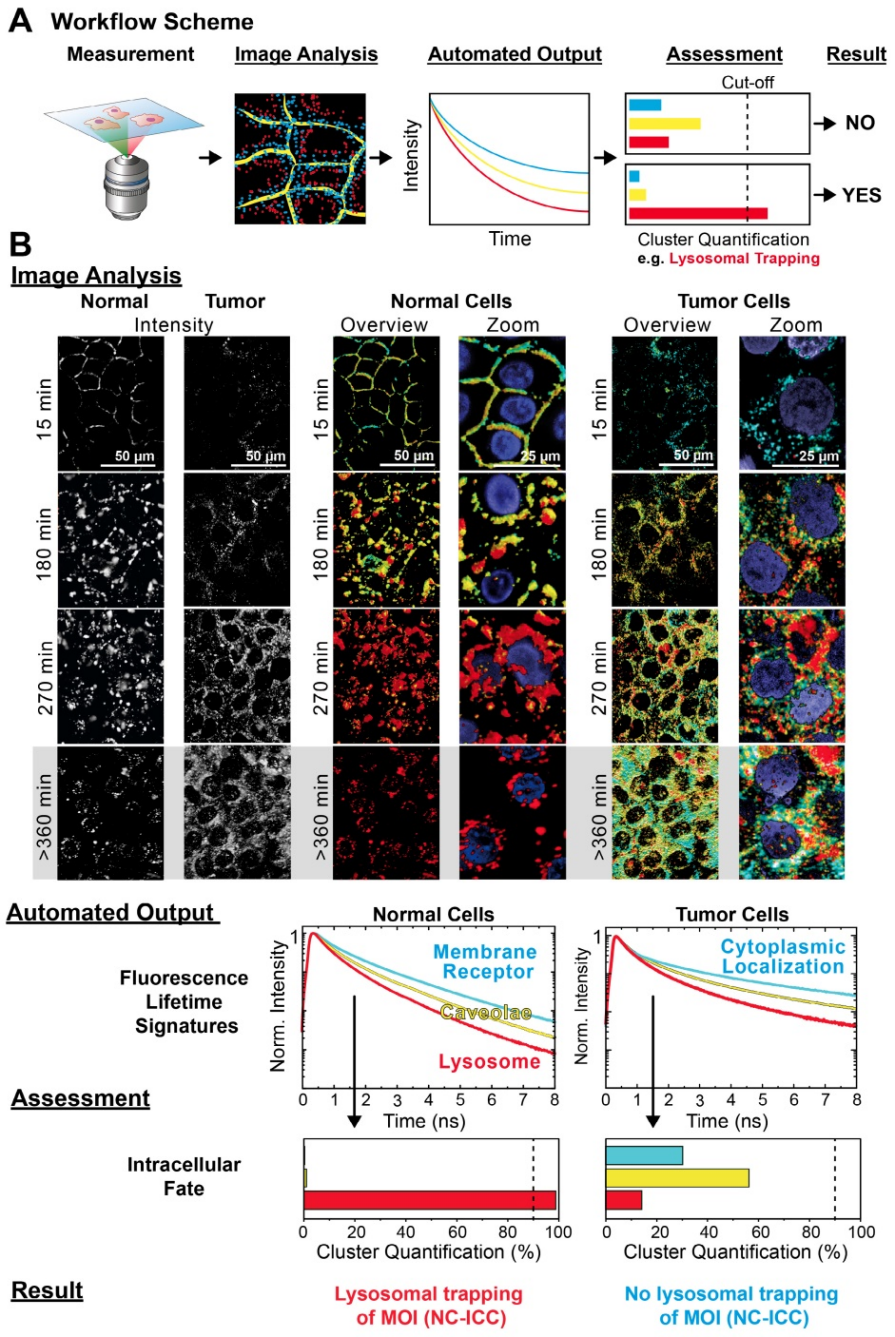
Automated MOI interactome assessment. (**A**) Workflow of automated Cluster-FLIM interactome assessment. (**B**) Image Analysis, Automated Output, Assessment and Result from the workflow as exemplified for the intracellular fate of MOI 2 (NC-ICC) in human keratinocytes and tumor cells (SCC-25). Experimental conditions as in Figure 1. Nuclei are stained by DAPI and shown in the zoomed images. Scale bars (overview): 50 µm; scale bars (zoom): 25 µm.

For the case at hand, Cluster-FLIM imaging and analysis proved the MOI 2 (NC-ICC) intracellular fate in lysosomes of normal human keratinocytes (**Figure 4**). As a next step, we unraveled the interactome differences of MOI 2 between normal human keratinocytes and tumor cells (SCC-25) by comparing the respective uptake time series (**Figure 1D, 5B**). The workflow as presented in **Figure 5A** enabled the image analysis to yield automated Cluster-FLIM results for application in high content screening.

For the *Image Analysis* (**Figure 5B**), we chose the same cluster feature space as for normal cells and color-coded the FLS as in the previous analysis, i.e., cyan for the fast, yellow for the intermediate, and red for the slowest decay signature (**Figure 1C**). In contrast to normal cells, the tumor cells displayed two significant differences from visual inspection: Firstly, no early membrane interaction of the NC-ICC was evident by the absence of a membrane localization of the cyan cluster (**Figure 5B**, 15 min incubation). Secondly, no lysosomal trapping was observed, but rather lysosomal escape and cytoplasmic localization occurred as seen by the diverse cluster appearance at extended incubation times (**Figure 5B**, > 360 min incubation).

The *Automated Output* provided the FLS and quantification of the respective clusters in the images (**Figure 5B**). The pre-defined cut-off in the *Assessment* (**Figure 5A**) directly enabled the retrieval of the *Result* from the Cluster-Quantification Plot. In our example, we performed the *Assessment* with a threshold of 90% for the red cluster identifying lysosomal appearance. While the relative appearance of the red cluster amounted to 99% in normal cells, the value was 14 % in tumor cells and therefore was well below the 90% threshold. According to the *Assessment* we obtained a negative result for lysosomal trapping as MOI 2's intracellular fate in tumor cells (**Figure 5B**).

Taken together, automated Cluster-FLIM analysis replaces the visual evaluation of FLIM images, thereby fulfills an essential requirement for high content screening and reduces analysis time. The *Image Analysis* of an image stack as provided in **Figure 5B** including *Automated Output* and *Assessment* required 12 s on a standard personal computer (3.4 GHz, 16 GB RAM (DDR3, 1600 MHz)) and can be further enhanced by advanced computer power. Bioinformatics 72 and machine learning tools empower the quantitative automated Cluster-FLIM assessment in large data sets, including cell segmentation tools 73, 74. In the future, the latter will allow correlating FLIM cluster localizations to the subcellular structures including the target. The detection of distinct differences in cellular uptake between normal and tumor cells are key in the design of tumor cell specific drug delivery systems with relevant trigger and drug release mechanisms 20, 75, 76.

**Figure 6:**
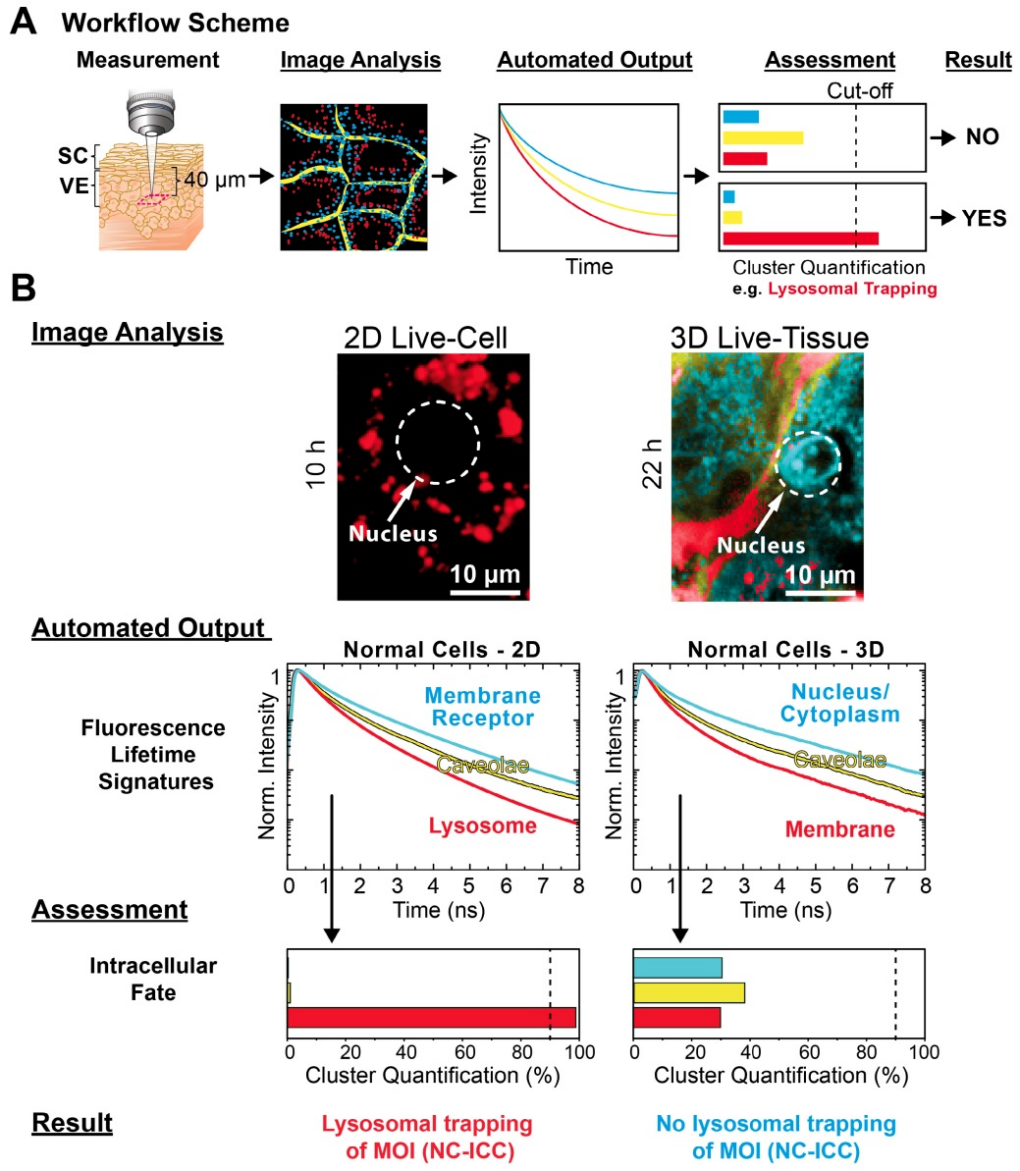
Application of automated MOI interactome assessment to 3D models. (**A**) Workflow of interactome assessment using mpFLIM in live tissue. (**B**) Image Analysis, Automated Output, Assessment and Result for the intracellular fate of MOI 2 (NC-ICC) in comparison between 2D cell culture and reconstructed human skin (3D tissue) using normal keratinocytes. Experimental conditions as described in Materials and Methods.

**Applicability of MOI interactome analysis to 3D models.** Since drug efficacy changes between assays in 2D cell culture and 3D tissues 3, 77, 78, we also investigated the MOI interactome in reconstructed human skin, a multilayered test platform with stratum corneum (SC), viable epidermis (VE), and dermis using mpFLIM 41, 43, 44. For analyzing the 3D interactome of the MOI 2 (NC-ICC), we compared the intracellular fate in 2D human keratinocyte culture with 3D tissue. According to the MOI 2 kinetics in 2D cell culture **(Figure 3B)**, the incubation time was set to 10 h in 2D cell culture and 22 h in the 3D tissue, respectively. The Cluster-FLIM workflow as shown in **Figure 5A** was adapted for the comparison to the 3D tissue (**Figure 6A**), thereby demonstrating the compatibility of Cluster-FLIM with innovate preclinical approaches and substantiating their benefit for drug development. Moreover, our Cluster-FLIM tool is easily adaptable to the analysis of other advanced FLIM implementations such as time-domain wide-field FLIM using lightsheet illumination 79.

For the *Image Analysis* (**Figure 6B**), we chose the same cluster feature space as for 2D cell culture (**Figure 1C**). In contrast to 2D culture, the epidermal cells displayed a significantly different spatial distribution of the clusters as easily seen from the Cluster-FLIM images in **Figure 6B**. Analyzing the intracellular fate of MOI 2, the *Automated Output* and *Assessment* revealed no lysosomal trapping in the epidermis, which is in contrast to the 2D culture. However, the yellow FLS in both model types showed a high similarity (**Figure 6B**), suggesting general internalization of the MOI 2, the dendritic core-multishell nanocarrier, via caveolae. In 3D, visual inspection revealed an accumulation of MOI 2 in cytoplasm and cell nuclei as the major site of intracellular fate. The difference in the intracellular fate is of relevance for predicted activity of the MOI in humans. This underlines the importance of new approaches including 3D cultures and cutting edge technologies such as Cluster-FLIM to study tissue penetration for reliable predictions of the fate in humans, which should increase the success rate in clinical trials 39, 43, 44, 80.

## Conclusions

High-content screening advances preclinical drug development by the fast and comprehensive analysis of complex models. The automated Cluster-FLIM outperforms current fluorescence microscopy and fluorescence lifetime image analyses in terms of information, contrast, spatial resolution, exposure times, and required fluorophore concentrations. Thereby, the Cluster-based FLIM method provides an enhanced insight into cellular processes including the accurate translation of fluorescence lifetime information into pharmacological pathways as shown for the cellular uptake of a nanocarrier as a molecule of interest. In this example, the intracellular fate markedly differed between 2D cell cultures and 3D tissues, emphasizing the need for qualified preclinical methods in drug development. This information was obtained as a direct outcome from the workflow for automated Cluster-FLIM, which can be transferred to any cell type and molecule of interest. By further combining the automated FLIM readout with bioinformatics and machine learning tools, Cluster-FLIM should become an essential part of high-content screening along the drug discovery pipeline to provide an unprecedented insight in target prediction and pathway profiling from the spatial FLIM interactome.

## Supplementary Material

Supplementary figures and tables.

## Abbreviations

FLIM: fluorescence lifetime imaging microscopy
MOI: molecules of interest
mp: multiphoton
HCS: high content screening
FLS: fluorescence lifetime signatures
NC: nanocarrier
Bodipy: 4,4-difluoro-1,3,5,7,8-pentamethyl-4-bora-3a,4a-diaza-s-indacene
CTB-A647: cholera toxin subunit B-Alexa Fluor™647
DAPI: 4′,6-diamidin-2-phenylindol
MβCD: methyl-β-cyclodextrin
Poly I: polyinosinic acid
Poly C: polycytidylic acid
ICC: indocarbocyanine
Cav-1-A488: caveolin-1-Alexa Fluor™488
PG: polyglycerol
NHS: N-hydroxysuccinimid
PEG: polyethylene glycol
SCC: squamous cell carcinoma
KGM: keratinocyte growth medium
DMEM: Dulbecco's Modified Eagle's Medium
PBS: phosphate buffered saline
TCSPC: time-correlated single photon counting
FWHM: full width half maximum
IRF: instrument response function
NHK: normal human keratinocyte
SR: scavenger receptor
ATP: adenosine triphosphate
BSA: bovine serum albumin
SC: stratum corneum
VE: viable epidermis
