# Supplementary material for: Faster, sharper, more precise: Automated Cluster-FLIM in preclinical testing directly identifies the intracellular fate of theranostics in live cells and tissue
Source: Theranostics. 2020 May 15;10(14):6322–36. doi: 10.7150/thno.42581 (PMC7255044; doi:10.7150/thno.42581)
Supplement: Supplementary file 1 — Supplementary figures and tables. [file thnov10p6322s1.pdf]

Supplementary Material

Faster, sharper, more precise: Automated Cluster-FLIM in preclinical testing directly identifies the intracellular fate of theranostics in live cells and tissue

Robert Brodwolf [†,Δ] , *Pierre Volz-Rakebrand* [†,Δ], *Johannes Stellmacher* [†, Δ], *Christopher Wolff* [∞], *Michael Unbehauen*[§], *Rainer Haag*[§], *Monika Schäfer-Korting* [∞], *Christian Zoschke* [∞] *and Ulrike Alexiev* [*,†]

[†] Institute of Experimental Physics, Freie Universität Berlin, Arnimallee 14, 14195 Berlin, Germany

[∞] Institute of Pharmacy (Pharmacology and Toxicology), Freie Universität Berlin, Königin-Luise-Str. 2+4, 14195 Berlin, Germany

[§] Institute of Chemistry and Biochemistry, Freie Universität Berlin, Takustr. 3, 14195 Berlin, Germany

*Correspondence and requests for materials should be addressed to U.A. (e-mail: ulrike.alexiev@fu-berlin.de)

Δ contributed equally

Supplementary Material

Table of Contents

Supplementary Figures

Figure S1	Page 3
Figure S2	Page 4
Figure S3	Page 7

Tables

Table S1	Page 9
Table S2	Page 10
Table S3	Page 11

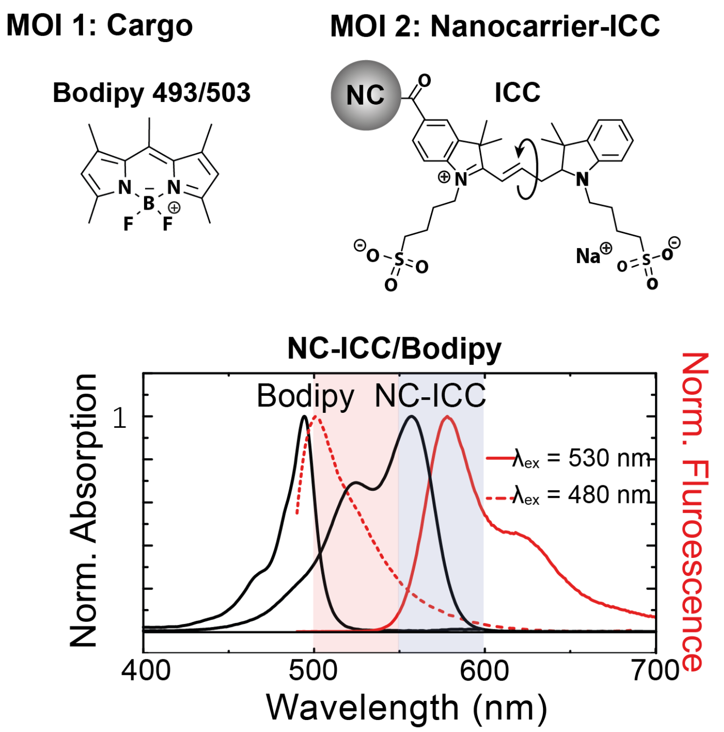

Figure S1. Normalized absorption (black) and fluorescence emission (red) spectra of Bodipy (MOI 1) and NC-ICC nanocarrier (MOI 2). Red and blue shaded areas in the spectra indicate the respective spectral range of emission filters used for Bodipy and NC-ICC, respectively. The structures of Bodipy and NC-ICC are shown.

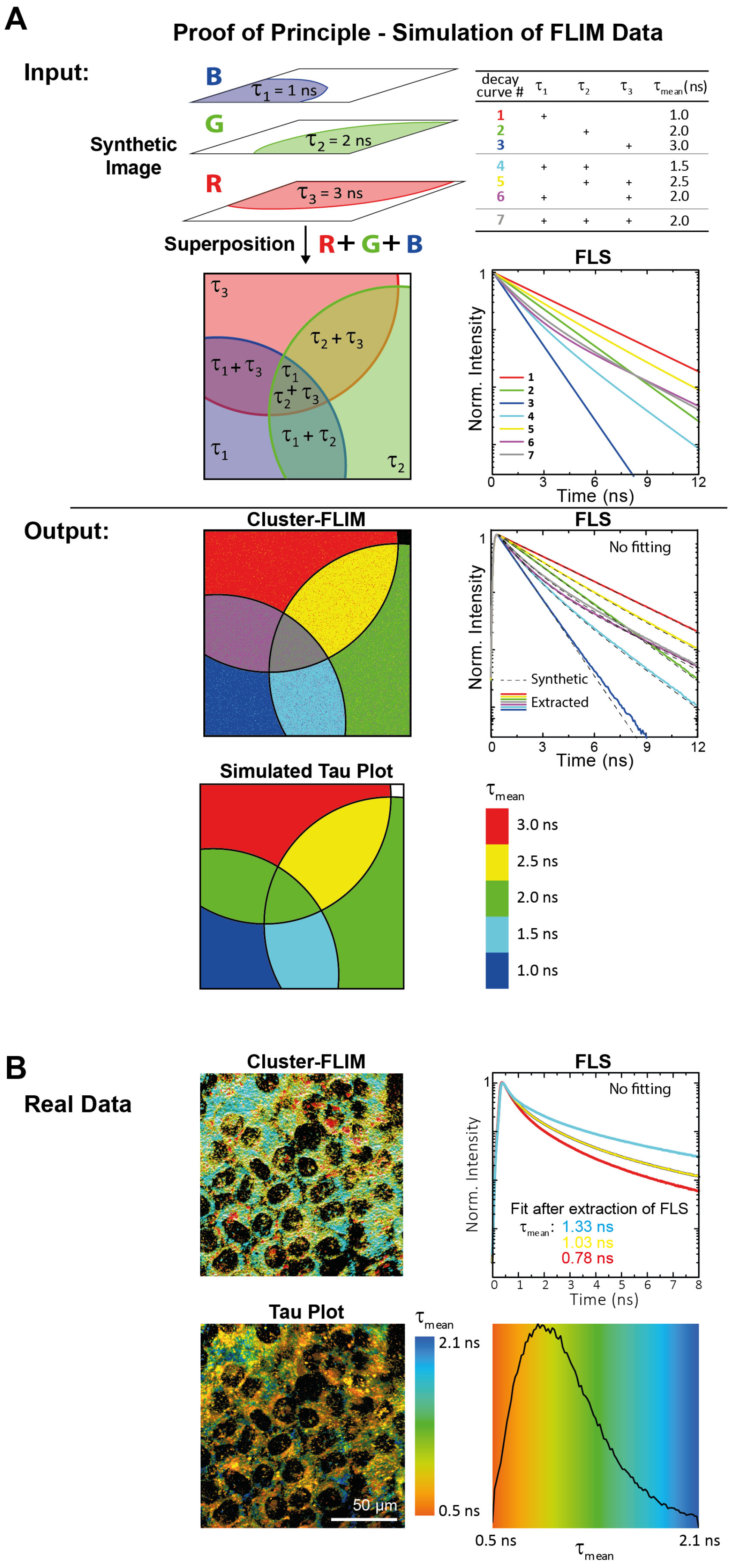
A
Proof of Principle - Simulation of FLIM Data
Input:
Synthetic Image
B
τ1 = 1 ns
G
τ2 = 2 ns
R
τ3 = 3 ns
Superposition
R+ G+ B
decay curve # τ1 τ2 τ3 τmean(ns)
1 + 1.0
2 + 2.0
3 + 3.0
4 + + 1.5
5 + + 2.5
6 + + 2.0
7 + + + 2.0
FLS
Norm. Intensity
Time (ns)
Output:
Cluster-FLIM
No fitting
Synthetic
Extracted
Simulated Tau Plot
τmean
3.0 ns
2.5 ns
2.0 ns
1.5 ns
1.0 ns
B
Real Data
Fit after extraction of FLS
τmean: 1.33 ns
1.03 ns
0.78 ns
Tau Plot
2.1 ns
0.5 ns
50 µm

Figure S2. Validation of Cluster-FLIM and proof of principle. (**A**) Generation of synthetic FLIM-data and Cluster-FLIM analysis for reliable discrimination of fluorescence decay curves with a high dynamic range. For the "Input" three layers of simulated FLIM-images (512 x 512 pixel) are generated and superimposed. In each pixel of the three circles monoexponential fluorescence decay traces are simulated with fluorescence lifetimes of τ_1=1 ns (B: blue), τ_2=2 ns (G: green) and τ_3=3 ns (R: red) with an average pixel intensity of 500 counts (cts) and Poisson distributed background noise. As in the experiment the theoretical fluorescence decay curve is convoluted with an IRF (here FWHM ~170 ps). In areas of overlapping circles the fluorescence decays traces are super-positions of the individual decays of R,G,B with equi-fractional contributions. This leads to seven different fluorescence decays signatures (including bi- and triexponential decay curves) in the seven areas separated by the colored boarders. Note, due to the superposition the pixels with the biexponential decay had an average pixel intensity of 1000 cts, and the pixels with triexponential decays had an average pixel intensity of 1500 cts, but resulting in a mean relative photon number of ~15% for each area of the 7 areas, with respect to the total photon number in the image. The "Output" of Cluster-FLIM clearly separates the seven FLS and reliably groups pixels with similar fluorescence decays curves in the image, whereas a Tau plot would only find 5 areas because area #2, #6, and #7 share the same mean fluorescence lifetime of 2 ns (see Table in the upper right corner, and green color in simulated tau plot). (**B**) Cluster-FLIM applied to real data (NC-ICC in SCC25 cells). Cluster-FLIM extracts three fluorescence lifetime cluster without the need for fitting the fluorescence lifetime traces. Each cluster is characterized by a unique fluorescence decay characteristics, represented by the FLS. The three extracted FLS are color-coded in cyan, yellow, and red (right). All image pixels belonging to the same cluster share the same decay characteristics and the false-color Cluster-FLIM image (left) is generated assigning a distinct color (corresponding to the FLS color) to all pixels belonging to the same cluster. Thus, the contrast in the Cluster-FLIM image is based on the

fluorescence decay shape in each cluster. Thereby, a high content and high contrast Cluster-FLIM image is generated and reveals the information enhancement compared to a conventional Tau plot.

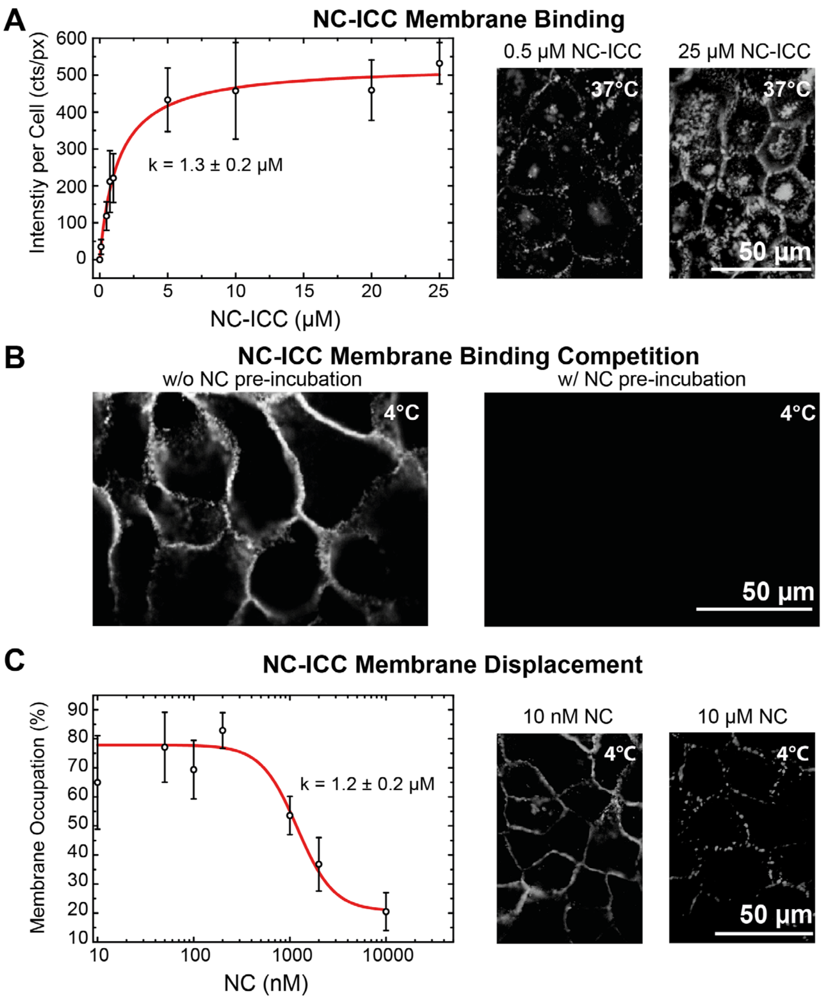

Figure S3. NC-ICC membrane interactions. (**A**) Binding of NC-ICC to normal human keratinocyte cell membranes as a function of NC-ICC concentration. The saturation of the NC-ICC binding curve points to a receptor-limited uptake mechanism. The non-linear binding behavior was fitted (equation (13) of main text), yielding a half-maximum uptake concentration (apparent receptor binding affinity) of 1.3 ± 0.2 µM NC-ICC. Values are mean ± SD (n=3-6). (**B**) Binding competition of NC-ICC with unlabeled NC. For binding competition keratinocytes were preincubated with 1000 µg/mL (50 µM) unlabeled NC for 180 min at 4 °C. Binding was performed with 10 µg/mL of NC-ICC without NC preincubation (left) and with NC preincubation (right). (**C**) NC-ICC displacement from keratinocyte cell membranes. NC-ICC (10 µg/mL, ~ 0.5 µM) was incubated in cell medium at 4 °C for 1 h. Afterwards unlabeled NC was added with concentrations ranging from 10 nM to 10 µM at

4 °C. At each NC concentration, the membrane area occupied by NC-ICC was calculated. The decrease of occupied membrane area with increasing NC concentration was fitted by a modified Hill function (equation (14) in main text), yielding a half-maximum binding constant of 1.2 ± 0.2 µM NC. The decrease in NC-ICC as a function of NC concentration is shown in the two images for 10 nM and 10 µM NC. Experimental conditions: NC-ICC with λ_{ex}=530 nm, λ_{em}=545-600 nm; objective: 60x water immersion.

Supplementary Material

Table S1. Fit parameters (equation (2) in main text) of the fluorescence decay traces (FLS) of the respective FLIM-clusters obtained by Cluster-FLIM of normal human keratinocytes after incubation with NC-ICC/Bodipy nanocarriers. τ_i: fluorescence lifetime of the *i*-th decay component in ns, α_i the corresponding relative amplitude in % (fractional amplitudes β_i shown in brackets), τ_m: population weighted mean fluorescence lifetime, $\tau_{m,a}$: amplitude weighted mean fluorescence lifetime, and χ^2_{red}: reduced chi square. Since the fluorescence decay traces are generated by integrating over all pixels of a FLIM-image the signal-to-noise is very high and the χ^2_{red} values are above 1. The quality of the fit is also judged from the residuals of the fit. Small deviations in the residuals become apparent at shorter times, probably due to deconvolution with the IRF. As the Cluster-FLIM analysis does not rely on fitting the fluorescence decay traces the fit data are only shown to give an estimate on the mean fluorescence lifetime. Experimental conditions: ICC (autofluorescence, cyan, yellow, and red cluster), λ_{ex}=530 nm, λ_{em}=545-600 nm; Bodipy (magenta cluster), λ_{ex}= 488 nm, λ_{em} = 500-550 nm.

Decay Species	α_1 (%) (β_1 (%))	τ_1 (ns)	α_2 (%) (β_2 (%))	τ_2 (ns)	α_3 (%) (β_3 (%))	τ_3 (ns)	α_4 (%) (β_4 (%))	τ_4 (ns)	α_5 (%) (β_5 (%))	τ_5 (ns)	χ^2_{red}	$\tau_{m,a}$ (ns)	τ_m (ns)
Autofluorescence	56.9 (27.4)	0.33	23.0 (26.2)	0.78	17.9 (38.1)	1.46	1.0 (2.9)	2.00	1.1 (5.5)	3.40	1.93	0.69	1.10
Slow (cyan)	48.5 (22.1)	0.43	21.5 (24.2)	1.06	21.0 (33.8)	1.52	7.1 (13.1)	1.74	1.9 (6.8)	3.40	3.1	0.94	1.33
Intermediate (yellow)	59.3 (33.3)	0.41	20.8 (25.1)	0.88	17.7 (35.6)	1.47	1.9 (4.2)	1.62	0.3 (1.8)	4.46	4.4	0.73	1.03
Fast (red)	37.8 (18.8)	0.28	42.4 (39.9)	0.53	12.8 (23.9)	1.05	6.8 (16.1)	1.33	0.2 (1.4)	3.91	2.8	0.56	0.78
Bodipy (magenta)	9.6 (2.9)	1.62	90.4 (97.1)	5.8	-	-	-	-	-	-	3.1	5.40	5.68

Table S2. Fit parameters (equation (2)) of FLS shown in Figure 2. τ_i: fluorescence lifetime of the *i*-th decay component in ns, α_i the corresponding relative amplitude in %. $\tau_{m,a}$: amplitude weighted mean fluorescence lifetime, and χ^2: reduced chi square. Experimental conditions: ICC, λ_{ex}=530 nm, λ_{em}=545-600 nm.

	FLS	α_1(%)	τ_1(ns)	α_2(%)	τ_2(ns)	α_3(%)	τ_3(ns)	α_4(%)	τ_4(ns)	χ^2_{red}	$\tau_{m,a}$(ns)
4 FLS<br>90 cts/px	Cyan	29.4	0.67	70.2	1.56	0.4	4.98	-	-	1.10	1.48
	Green	53.3	0.17	27.4	0.54	14.3	1.00	5.0	1.56	1.06	0.78
	Magenta	76.1	0.22	19.1	0.53	4.5	1.07	0.3	1.17	2.67	0.46
	Red	94.9	0.15	4.5	0.33	0.6	0.9	-	-	1.14	0.20
5 FLS<br>170 cts/px	Cyan	34.9	0.78	64.8	1.65	0.3	6.29	-	-	1.39	1.53
	Green	56.7	0.25	32.4	0.77	6.9	1.14	4.0	1.85	1.74	0.83
	Magenta	68.5	0.19	23.8	0.43	7.2	0.87	0.5	1.76	1.34	0.46
	Red	94.8	0.16	4.4	0.40	0.8	0.87	-	-	1.74	0.24
	Yellow	56.5	0.33	29.6	0.97	13.6	2.11	0.3	4.42	1.17	1.30

Table S3. Inhibition of cellular uptake of NC-ICC nanocarrier towards NHK after 180 min using low temperature, ATP and cholesterol depletion by azide and MßCD, respectively. Blocking of caveolin phosphorylation, macropinocytosis (PI3K activity), and clathrin mediated endocytosis was performed by addition of genistein, wortmannin, or chlorpromazine, respectively. Scavenger receptor class A ligands polyionosinic acid and fucoidan were used to inhibit NC-ICC binding to scavenger receptors. Poly C is not a ligand of scavenger receptor and utilized as a positive control. The 37 °C uptake measurement was used as control reference and set to 100%.

Inhibitor	Concentration	NC-ICC within NHK (% of control ± SD)
Low Temperature (4°C)	-	11 ± 2
Azide	3 mg/ml	0.5 ± 0.5
MβCD	5 mg/ml	13 ± 6
Genistein	27 µg/ml	10 ± 5
Polyinosinic acid (Poly I)	50 µg/ml	8.6 ± 2.3
Polycytidylic acid (PolyC) (positive control)	50 µg/ml	98 ± 14
Fucoidan	100 µg/ml	0.9 ± 0.7
Wortmannin	150 ng/ml	105 ± 25
Chlorpromazine	10 µg/ml	77 ± 8
